# Knowledge and Use of Folic Acid among Women Attending the high-Risk Prenatal Clinics at the Adults University Hospital in Puerto Rico

**DOI:** 10.23937/2474-1353/1510043

**Published:** 2016-12-14

**Authors:** Stephanie Rivera-Segarra, Lizzie Ramos-Tollinchi, Natalia Cárdenas-Suárez, Josefina Romaguera

**Affiliations:** Department of Obstetrics and Gynecology, University of Puerto RicoSchool of Medicine, Puerto Rico

**Keywords:** Folic acid supplementation, Prenatal care, Neural tube defects, Unplanned pregnancies

## Abstract

**Introduction:**

Pregnancies affected with neural tube defects (NTDs) are mostly associated to maternal deficiency of folic acid (FA). Although supplementation is recommended for all women of childbearing age, the incidence of NTDs in Puerto Rico has not shown a significant decrease.

**Objective:**

The goal of this study was to assess the awareness and level of knowledge of FA supplementation among women attending prenatal clinics, and correlate this knowledge with the source ofinformation and the actual use of FA. A secondary objective was to corroborate or abrogatethe association of the lack of FA supplementation with the occurrence of unplanned pregnancies.

**Methods:**

This descriptive study was conducted at the High-Risk Prenatal Care Clinicsof the Adults University Hospital from August 2015 to November 2015. The answers to a non-validated self-administered questionnaire were assessed and then analyzed with Epi Info 7.

**Results:**

From a total of 200 Hispanic female participants, 87.0% were Puerto Rican, most (69.0%) had an education above high school level and 54.5% had a low-income status. Overall, 66.5% were taking FA at the time of the interview, 77% understood that the best time to start FA supplementation was prior to conception, but only 23% of the participants actually began preconceptional FA intake. Unplanned pregnancies were reported in 70.5%. Most referred to have received information about FA benefits from a healthcare professional, yet many could not identify all of FA benefits.

**Conclusion:**

Although most participants were aware of the best time to begin FA supplementation, the majority began intake once pregnancy was discovered; timing related to the 70.5% unplanned pregnancies. Information received is not sufficient sincemost women are not entirely clear about the benefits of FA supplementation, despite their source of information. In caring for women of childbearing age, further investigation is required to optimize educational strategies and methodologies.

## Introduction

Folic acid (FA), a water-soluble B vitamin, is well-known to be essential for several bodily functions and for the growth of the embryo’s spinal cord. Accordingly, FA has been recognized as an important supplement during the early stages of fetal organogenesis, establishing a link between FA supplementation and diminished birth defects [1]. This vital role of FA in the healthy formation of the embryo and fetus has been further established by the many studies demonstrating that preconceptional FA supplementation leads to the decline of neural tube defects (NTDs) [1-4], cleft lip and/or palate [5], early spontaneous preterm birth [6] and more recently congenital heart defects [7]. Although its exact mechanism is unknown, the increasing evidence of preconceptional FA supplementation reducing the incidence of birth defects led to the mandatory law enforcement, in 1988, of fortification of breads and other grains with FA, especially since the average dietary consumption of FA in women was lower than the recommended amount [8].

After the fortification of the food supply, studies have shown a relative increase in the FA serum levels in women in their reproductive age and a corresponding decrease in the incidence of NTDs [8,9]. Current recommendations stipulate that all women of childbearing age intake a supplement of 400 mcg daily of FA as the minimum dose required to avert NTD-affected pregnancies [10]. Unfortunately, general FA supplementation still remains low in women despite public campaigns, education, and government-mandated fortification of foods. According to Pregnancy Risk Assessment Monitoring System (PRAMS), FA consumption in women was highest among other race/ethnicity (33.0%), followed by Whites (32.4%), Blacks (19.5%) and Hispanics (10.9%) [11]. This is of great importance considering the incidence rates of NTDs are known to vary by race and Hispanics have the highest rates [12]. The decline in the prevalence of NTDs have been found to be more significant in countries where FA supplementation has impacted the majority of the women of childbearing age, leading to a decrease in approximately 70% of annual cases when FA intake begins prior to conception [13]. In contrast, the annual decline in the prevalence of NTDs in Puerto Rico is merely 3.5% [14]. The reasons for this minimal reduction include: the lack of awareness of the benefits of FA supplementation in Puerto Rican women of childbearing age, the high rate of unplanned pregnancies (64.9%) and, correspondingly, the lack of early prenatal care [1,15]. For these reasons alone, there is an undeniable need for having a knowledgeable and wide spread source of education regarding FA supplementation and pregnancy planning in Puerto Rico.

The overall goal of this study was to assess the awareness and level of knowledge of FA supplementation among Puerto Rican women attending the high-risk prenatal clinics at the Adults University Hospital, and correlate this knowledge with the source where the information was obtained. A secondary objective was to corroborate or abrogate the association of the lack of FA supplementation with the occurrence of unplanned pregnancies.

## Methods

The study was conducted at the high-risk prenatal care clinics of the Adults University Hospital, University of Puerto Rico School of Medicine, Medical Science Campus. A non-validated, self-administered questionnaire was offered to consenting adult women (21 years and older) during the months of August 2015 through November 2015. The 60-item questionnaire is divided into several sections focusing each on the assessment of patient demographics, past medical history, obstetrical history, and use/knowledge of FA. Inclusion criteria for the study consisted of: women aged 21 years and older, and being pregnant or less than six (6) weeks postpartum at time of questioning. The only exclusion criteria were: having a diagnosis of epilepsy and/or receiving anti-epileptic treatment. Those agreeing to participate were escorted to a private room for the informed consent process with the presence of a trained healthcare professional (nurse, physician or investigator) available to answer questions if necessary. All participants signed the written consent prior to completing the questionnaire. After completion, the questionnaires were duly secured for confidentiality purposes and the required protection of personal health information. All collected data was converted into random number identifiers to maintain anonymity and coded to facilitate analysis using the EpiInfo 7 statistical software. The analysis focused on evaluating the occurrence of events regarding the examined population. Frequencies were used to determine the percentages of women: (a) taking FA, (b) with unplanned versus planned pregnancies, (c) with knowledge of FA supplementation, and (d) aware of preconceptional FA supplementation.

## Results

### Sample for analysis

Of a total of 233 recruited participants, 33 needed to be excluded because 16 had exclusion criteria and 17 answered the questionnaire in an incorrect manner. Thus, the final sample amenable for statistical analysis consisted of 200 verified and validated questionnaires.

### Demographics and pregnancies

All participants disclosed to be of Hispanic ethnicity, being Puerto Ricans the biggest group with 87.0%. Most participants (69.0%) indicated having an education above high school level. In comparison, participants with only a High School diploma were a 27.0% and only a 4.0% did not completed High School. Participants self-reported a low annual household income, with most of the participants (54.5%) reporting having an annual household of less than $20,000. A group of 23.0% of participants reported the current pregnancy as their first, while 54.5% reported having one or two prior pregnancies. However, the great majority (70.5%) reported the current pregnancy as unplanned (Table 1).

### Folic acid intake

Among all participants, 66.5% indicated taking FA at the time of the interview and 77.5% understood that the best time to start FA supplementation was before pregnancy. However, only 23.0% reported taking FA prior to conception and 59.0% began supplementation once they discovered to be pregnant. Of those actively taking FA, 70.5% reported this intake was due to medical recommendation (Table 2).

### Acquisition of knowledge about folic acid

**Figure 1:**
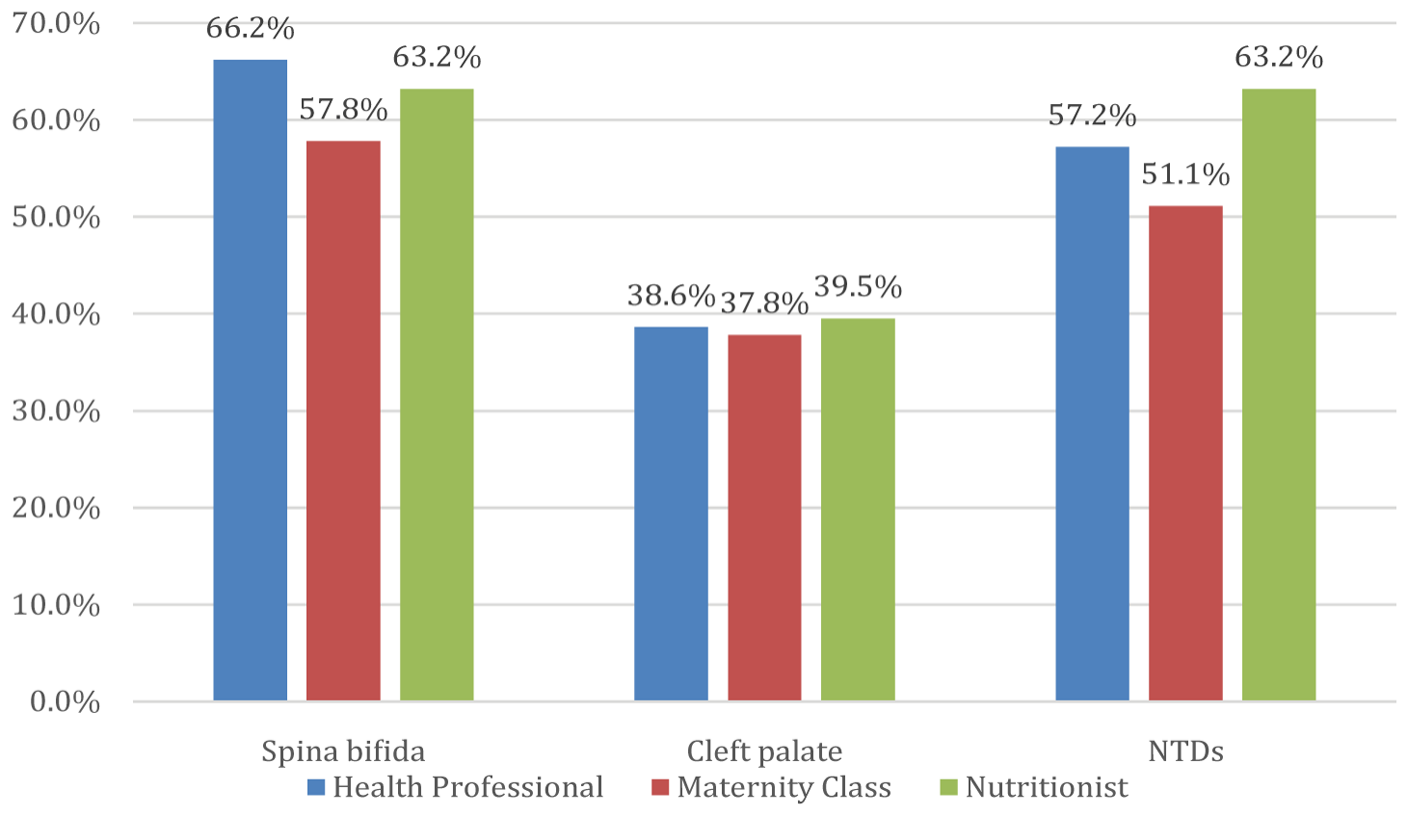
Summary of assertively identified benefit over condition.

The options offered in the questionnaire as possible sources of information for gaining knowledge of FA supplementation were: (a) healthcare professionals (physicians/nurses), (b) maternity classes, (c) nutritionists, (d) newspapers/magazines, (e) friends/family members, and (f) television. Although most participants selected more than one source of information, each option was evaluated individually. The most selected options were: (a) healthcare professionals (physicians/ nurses) for 72.5%, (b) maternity classes for 22.5%, and (c) nutritionists for 19.0% (Table 3). Further analysis was focused on these top three options. Of the women selecting healthcare professionals as their source of information, 66.0% could identify that FA intake could prevent the development of spina bifidain particular, 57.2% identified prevention of NTDs in general and 38.6% identified prevention of cleft lip and/or palate. Of the women selecting maternity classes as their source of information, 57.8% identified spina bifida, 51.1% identified NTDs, and 37.8% identified cleft lip and/or palate. Of the women who selected nutritionists as their source of information, 63.2% identified both the prevention of spina bifida in particular as well as NTDs in general, and 39.5% identified cleft lip and/or palate (Figure 1).

## Discussion

The evidence that consumption of FA before conception (preconceptional) and during early pregnancy (periconceptional) can reduce the number of NTDs has been accumulating for several years now. From a public health perspective, the delivery of FA to the general population consists of three approaches: a) improvement of dietary habits, b) fortification of the food supply, and c) use of dietary supplements. After the government-mandated fortification of foods with FA took effect, studies have shown the benefit of reducing the incidence of NTDs [1,8]. With hopes to continue this positive trend, the US Preventive Task Force (USPTF) has recommended that women of childbearing age consume 400 mcg of FA as a dietary supplement at least 1 month prior to conception [4]. It is known that a large portion of the mother’s stores of FA is utilized during the embryo’s development, specifically during organogenesis, which is mostly completed by the 8^th^ week of gestation. This is the main reason why women should make sure to have adequate body stores of FA prior to conceiving. Unfortunately, the wide spread lack of pregnancy planning hinders these recommendations and offers a challenge for optimal preconceptional FA supplementation.

Specifically in Puerto Rico, unplanned pregnancies are a significant problem. De la Vega, et al. reported in 2002 a study that found 64.6% of the pregnancies to be unplanned and merely 54.6% of the women took preconceptional FA supplements, although 87.7% disclosed to be aware of the importance of FA [3]. In comparison, this study (13 years later) reports a relative 5.6% increase in unplanned pregnancies, a relative 31.6% decrease in preconceptional FA supplementation, and a relative 10.2% decrease in awareness of FA importance. This disproportionate and significant decrease in women actually taking FA supplements prior to conception is worrisome and cannot be explained alone by a slight increase in unplanned pregnancies and a mild decrease in awareness. However, in terms of the women actively taking FA supplements at the time of the interview, the findings in this study (66.5%) were found to be similar to those found in a study reported by Khodr, et al. [10].

As to pregnant women correctly identifying the main benefits of FA supplementation, this study found that most could identify the prevention of spina bifida in particular, NTDs in general, and less frequently cleft lip and/or palate. However, only a low percentage of participants could name all benefits. This inability to identify all benefits could be particular to this population sample that has a low education level and low income status, which suggests a gap in their knowledge of the subject.

On further analysis, the identified benefits of FA were subdivided according to their selected source(s) of information. Those that received information from a healthcare provider and/or maternity class more frequently identified prevention of spina bifida as a beneficial effect of FA supplementation. On the other hand, those that received their information from a nutritionist identified both the prevention of spina bifida in particular and NTDs in general as benefits. The fact that both options resulted in an equal percentage might indicate non-discrimination of the options. Of note, the benefit of prevention of cleft lip and/or palate was identified significantly less when compared to the other two options and irrespective of the source of information. This finding suggests a gap in the content of information transmission from all sources as well. Thus, both the source and the content of information are important factors to take into account when studying the adherence of FA supplementation in women of childbearing age.

Regarding preconceptional FA supplementation, a significant disparity between knowledge and implementation was found. Although the great majority of women were aware that FA supplementation should begin prior to conception in this study, less than a fourth of them actually implemented the intake. Again, the low education level among the participants could play a role in the disparity. However, this disparity has also been shown in other studies finding that knowledge of recommendations is, actually, a poor predictor of adherence to nutritional supplementation [16]. On the other hand, the fact that this study group merely had 22.5% planned pregnancies, only these women had the best opportunity to comply with the recommended preconceptional FA intake. Both Crider and De la Vega [1,3] have suggested that high rates of unplanned pregnancies could possibly contribute to the increasing prevalence rates of NTDs. Nevertheless, to fully understand why the majority of women continue with unplanned pregnancies and low adherence of preconceptional FA supplementation requires further investigation.

## Conclusion

In this study, the sample of women interviewed in our clinics shown most pregnancies to be unplanned and FA supplementation not preconceptional as recommended by the USPTF [4]. Most women were able to identify the main beneficial effects of FA but not all of them. These findings demonstrate that the knowledge of FA supplementation of our particular sample is incomplete, raising it as one of the possible factors that affect FA supplementation adherence. Further investigation is required to better understand all factors leading to unplanned pregnancies and to better optimize educational strategies and methodologies in regards to preconceptional care of women of childbearing age.

## Figures and Tables

**Table 1:** Demografics.

**Nationality**	
*Puerto Rican Nationality*	87.0%
**Education**	
*Less than high school education*	4.0%
*Hight school diploma*	27.0%
*Above high school education*	69.0%
**Economic Status**	
*From 0 to $19,999 annual income*	54.5%
*From $20,000 and above annual income*	11.0%
*Refused to answer*	34.5%
**Number of Pregnancies**	
*First pregnancy*	23.0%
*Second or More*	54.5%
**Unplanned pregnancies**	70.5%

**Table 2:** Knowledge and use of Folic Acid.

Knowledge of FA prior to pregnancy	77.5%
Preconceptional of use of FA	23.0%
Began use of FA after pregnancy was discovered	59.0%
Actively taking FA at the time of interview	66.5%

**Table 3:** Identified source of information for FA knowledge

Health professional	72.5%
Maternity classes	22.5%
Nutritionist	19.0%
Friend or Family members	15.5%
Newspaper or Magazine	10.0%
TV	8.5%

Participants could identify more than one source of information.
